# T cell assays for non-clinical immunogenicity risk assessment: best practices recommended by the European Immunogenicity Platform

**DOI:** 10.3389/fimmu.2025.1723110

**Published:** 2026-01-12

**Authors:** Sophie Tourdot, Anette Christine Karle, Marc Rosenbaum, Chloé Ackaert, Pauline Le Vu, Michael Gutknecht, Maryam Ahmadi, Annelies W. Turksma, Timothy P. Hickling

**Affiliations:** 1 Pfizer Inc., Research and Development, Immunogenicity Sciences, Andover, MA, United States; 2 Novartis Pharma AG, Biomedical Research Center, Immunogenicity and Mechanistic Immunology, Basel, Switzerland; 3 Hexal AG (a Sandoz Company), Global Clinical Development, Clinical Bioanalytics, Holzkirchen, Germany; 4 IQVIA Laboratories, In vitro Immunology (ImmunXperts SA), Gosselies, Belgium; 5 Abzena Ltd., Bioassay Department, Cambridge, United Kingdom; 6 Immunotherapy Center Amsterdam University Medical Centers (UMC), Amsterdam, Netherlands; 7 Roche Pharma Early Research and Development, Cambridge, United Kingdom

**Keywords:** immunogenicity, non-clinical risk assessment, T cell assays, best practices, de-immunization, mitigation

## Abstract

*In vitro* and *in silico* tools help drug developers reduce unwanted immunogenicity of biologics at the design stage. These include assays that examine different immune system processes leading to anti-drug antibody (ADA) or cytotoxic cellular response development, such as activation and peptide presentation by antigen-presenting cells, and CD4+ or CD8+ T cell activation, proliferation, and specificity. The CD4+ T cell response is critical for establishing persistent, class-switched and affinity-matured ADA that are more likely to have a clinical impact. Various formats of CD4+ T cell assays raise concerns about quality, variability, and validity across laboratories. Harmonization on some key aspects of these assays is achievable, although full standardization among industry and academic labs is unlikely. Thus, the European Immunogenicity Platform Non-Clinical Immunogenicity Risk Assessment working group (EIP-NCIRA) sought to establish good practices to maximize data confidence and ensure consistent data interpretation within each assay format. The recommendations presented regard key assay parameters that will better ensure consistency across the field including donor selection, cell and test article quality control, data analysis, as well as implementation of standard controls to further reduce analytical variability.

## Section 1 - Introduction

**Figure 1:**
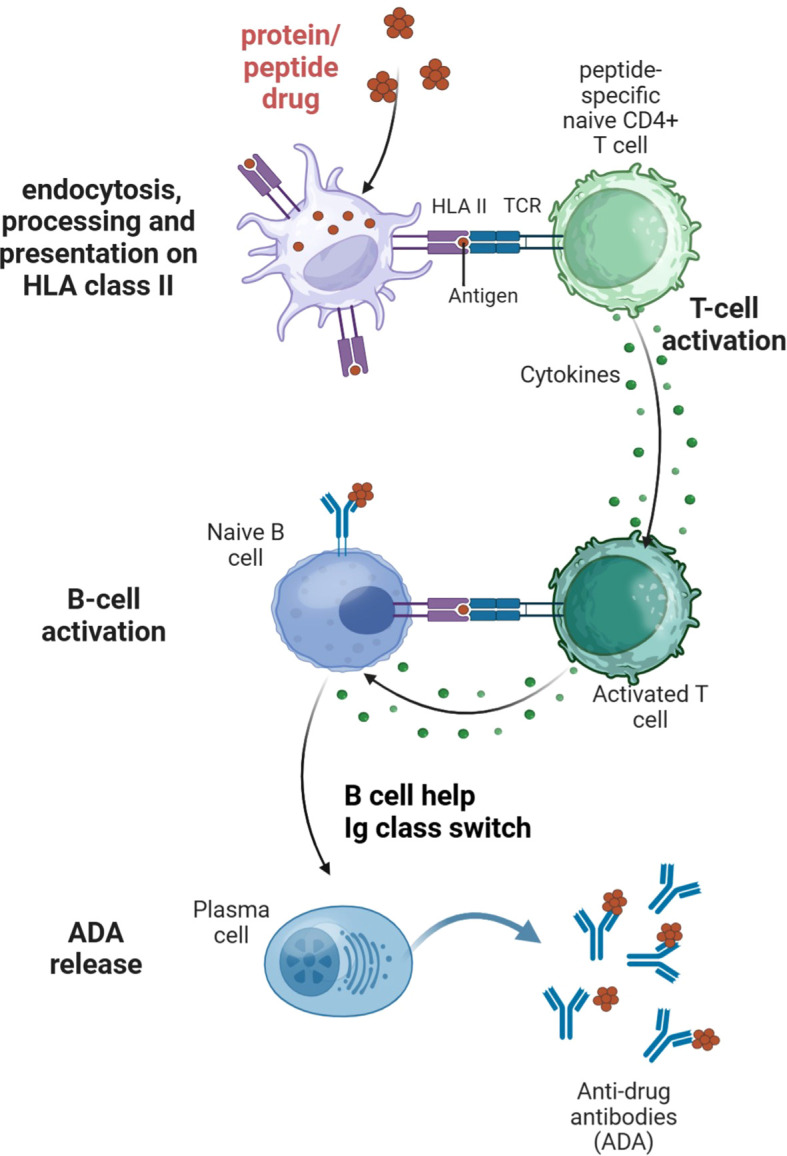
Antigen presenting cells, T and B cells interplay leading to anti-drug antibody development. Peptide and protein therapeutics are taken up and processed by professional antigen presenting cells (APCs). Peptides are bound to specific HLA class II molecules, with peptide:HLA complexes presented on the surface of the APC. A T cell with the corresponding peptide-specific T cell receptor can bind to the peptide:HLA complex. This recognition and additional interaction of co-stimulatory molecules lead to activation of the T cell and cytokine secretion. Activated T cells interact with B cells presenting the same peptide:HLA complex, leading to B cell activation, Ig class switch, and the release of anti-drug antibodies (ADAs). Adapted from Karle et al., 2025. Created in BioRender. Tourdot, S. (2025) https://BioRender.com/w5b0i4u.

The study and mitigation of unwanted immune responses to therapeutic peptides, proteins and other large molecule drugs is still an evolving science. Such immunogenicity is most frequently measured as ADA. Root cause analysis of immunogenicity commonly identifies several contributing factors, which can be related to three key cell types of the immune system, namely professional antigen presenting cells (APCs), T cells, and B cells (**Figure 1**). Evaluating the immunogenicity risk of a new biologic involves determining the likelihood of inducing ADA as well as an assessment of possible consequences of these ADA (1–4).

Prior to clinical studies, there are a few options to assess the potential for unwanted immune responses to a drug; *in silico* models which can be used as stand-alone tools or applied to guide human peripheral blood mononuclear cells (PBMC)-based *in vitro* experiments (5–7). Of note, *in vivo* studies with preclinical species are generally recognized as having limited value for predicting ADA incidence (8), but the consequences of immunogenicity on drug pharmacokinetics and safety observed in these studies might be relevant to inform the next steps of drug development (9). Amongst the community of scientific experts in the field of immunogenicity, T cell assays have become valuable for candidate selection and decision-making during drug discovery and development as T cells are a key prerequisite for the generation of robust ADA responses by collaborating with B cell populations (10, 11). T cell assays can be used to look at overall immunogenicity potential intrinsic to the drug product or impurities and can support the choice for a drug candidate to move forward to clinical development. For some new modalities, such as gene therapy, assessing the potential for CD8+ T cell-mediated cytotoxic activity, which can result in direct clinical consequences (hypersensitivity, killing of transfected cells), is also warranted and has been described elsewhere (12). CD8+ T cell risk assessment assays are out of the scope of this paper.

Several T cell assays have been independently developed with a variety of formats and protocols (13), with an extensive review of the assays conducted in 2020 (14). Here, examples to illustrate the main categories of T cell assay formats are presented in **Table 1**. The assays are often tailored to the specific needs of the laboratories, based on origin and type of blood source, infrastructure/equipment, or the need to conduct them in conjunction with other assays. The interpretation of assay results is therefore currently limited by the lack of established standards.

**Table 1:** Overview of *in vitro* T cell assay formats.

Assay format	Readout	Examples	Description	Test article
DC:T assay (long-term)	IFN-γ secretion	Quantitative analysis of the CD4+ T-cell repertoire specific to therapeutic antibodies in healthy donors (28)	Long-term DC:T assay (exceeding 2 weeks including moDC generation) to identify CD4+ T cell epitopes and analyze antigen-specific CD4+ T cell repertoire. Typically, 1:10 DCs: CD4+ T cells ratio	Whole protein for T cell line generation; whole protein or peptides for recall
DC:T assay (short-term)	^3^H-thymidine incorporation (proliferation) and IL-2 secretion	Secukinumab, a novel anti–IL-17A antibody, shows low immunogenicity potential in human *in vitro* assays comparable to other marketed biotherapeutics with low clinical immunogenicity (23)	Short DC:T assay (2 weeks including moDC generation) to assess T cell responses against protein antigens	Whole protein
	IFN-γ secretion	Validation of a Dendritic Cell and CD4+ T Cell Restimulation Assay Contributing to the Immunogenicity Risk Evaluation of Biotherapeutics (27)		
	CFSE and EdU staining (proliferation) and IFN-γ secretion	Preclinical immunogenicity risk assessment of biotherapeutics using CD4+ T cell assays (24)		
	IFN-γ secretion	Secukinumab Demonstrates Significantly Lower Immunogenicity Potential Compared to Ixekizumab (30)		
Whole PBMCs assay	IFN-γ, TNF-α and IL-2 via flow cytometry	A T-cell-based immunogenicity protocol for evaluating human antigen-specific responses (31)	Long-term PBMC -based assay to investigate peptide-specific T cell responses	Peptides
	IFN-γ secretion	Quantitative analysis of the CD4+ T cell response to therapeutic antibodies in healthy donors using a novel T cell:PBMC assay (29)	Short-term PBMC- based assay: isolated CD4+ T cells co-cultured with irradiated autologous PBMCs	Whole protein
	Activation-induced markers (AIM) and Ki-67 (proliferation)	Immunogenicity risk assessment for biotherapeutics through *in vitro* detection of CD134 and CD137 on T helper cells (26)	Short term PBMC- based assay identifying early changes in activation markers in response to antigen recall	Whole protein
CD8+-depleted PBMCs assay	CFSE (proliferation)	*Post-hoc* assessment of the immunogenicity of three antibodies reveals distinct immune stimulatory mechanisms (25)	Short CD8+-depleted PBMC assay to assess T cell responses against protein antigens	Whole protein or peptides

**Figure 2:**
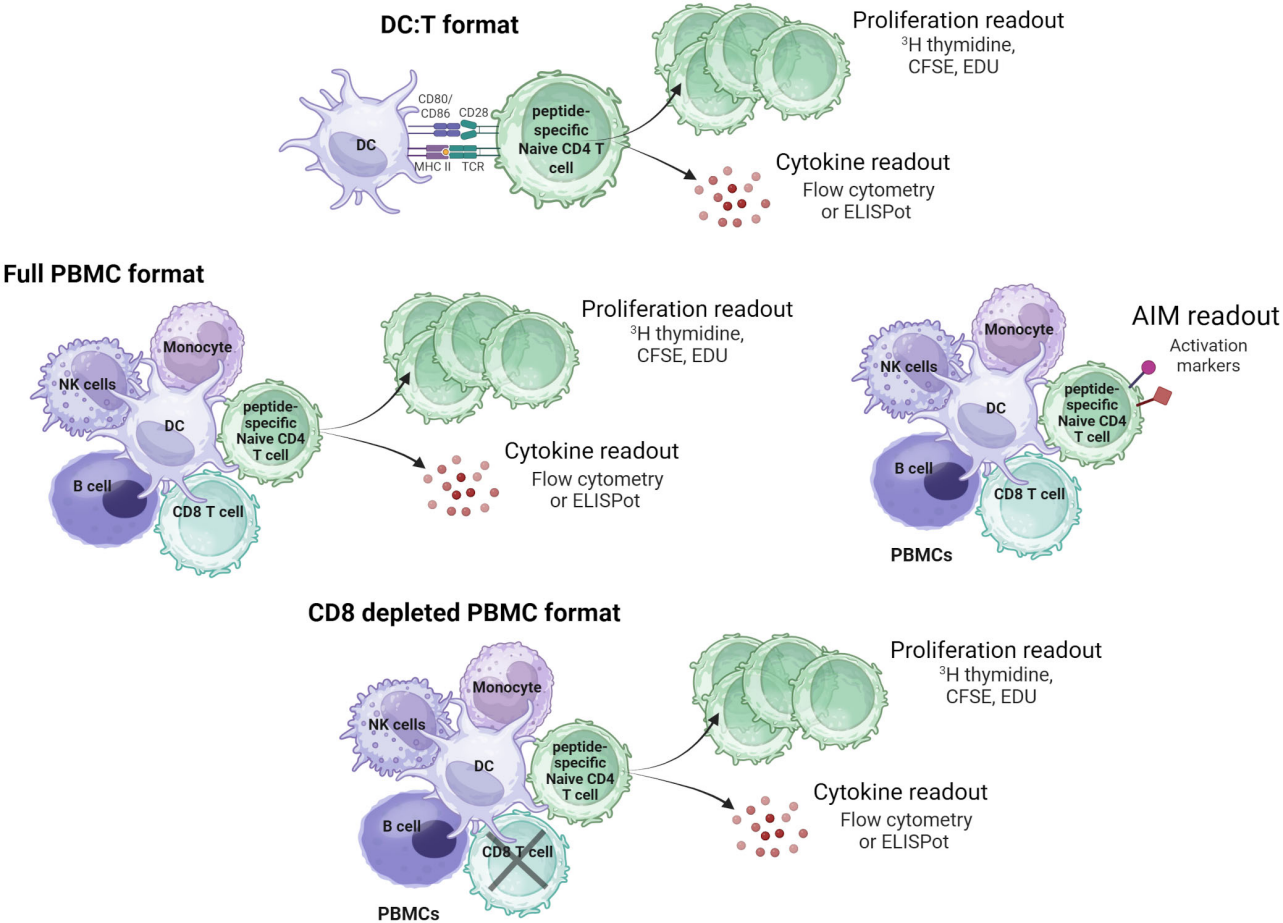
T cell assay formats and readouts. The DC:T cell format is the simplest format in terms of the cell types present, typically measuring T cell proliferation or activation as readouts. In contrast, PBMC-based assay formats include a variety of immune cell types, all of which may contribute to the overall immune response. Both the full PBMC and CD8+-depleted PBMC format use similar readouts to the DC:T cell assay format. The AIM assay is performed using the full PBMC format, with activation markers serving as readout. Created in BioRender. Karle, A. (2025) https://BioRender.com/8l8rsts.

We aimed to establish best practices to help address these limitations. The recommendations include donor selection, cell and test article quality control, readouts, data analysis, and the implementation of standard controls to reduce analytical variability. Most of the best practices outlined here apply to all NCIRA *in vitro* cell-based assays, and some are specific to T cell assays. It is important to note that here we review and offer guidance on APC-based T cell assays, as illustrated in **Figure 2**. It is key to emphasize that the EIP-NCIRA does not make recommendations on which specific assay formats to use or the brands of reagents and consumables.

## Section 2 - Overview of *in vitro* T cell assay formats

T cell assays are frequently employed to accomplish (i) the design of molecules with reduced immunogenic potential by identifying and removing foreign CD4+ T cell epitopes, (ii) *post-hoc* mechanistic analyses using patient samples upon clinical observation of ADA responses and (iii), establishment of *in vitro in vivo* correlations (IVIVC). An example of immunogenicity mitigation at the design stage is the work by Winterling et al. (15). The administration of blood coagulation factor VIII (FVIII) is an effective treatment for patients with Hemophilia A; however, its pronounced immunogenicity poses a significant challenge to therapeutic efficacy (16). In this study, **Table 1** Winterling and colleagues aimed to engineer a FVIII variant with a lower immunogenic sequence risk. The authors utilized *in silico* methodologies to identify CD4+ T cell epitopes and determine suitable amino acid substitutions to eliminate these epitopes. Subsequently, an *in vitro* DC–T cell assay conducted with healthy donor PBMC was performed to verify the effects of the introduced mutations. The results showed that most donors exhibited a decrease in CD4+ T cell proliferation responses to the de-risked variant relative to native FVIII, with reductions as high as 50-60%. Importantly, the engineered variant retained comparable functional activity to that of the native FVIII.

Several publications report the use of T assays to decipher mechanisms underlying the development of ADA (17–19). As mentioned earlier, ADA can impact patient safety. An illustration of the use of T cell assays to investigate a possible role of ADA development in the occurrence of adverse events in patients treated with brolucizumab has been reported. Karle et al. aimed to facilitate root cause analysis of adverse clinical effects in brolucizumab patients by determining whether drug-reactive T cells are present in the blood of treated patients, and whether differences exist in the immune response between patients with and without adverse effects. The study demonstrated that significant total and memory T cell responses were observed exclusively in patients experiencing adverse events. These findings indicate that individuals treated with brolucizumab who possess pre-existing memory against the drug may develop T cell reactivity and subsequent ADA upon re-exposure to brolucizumab (20).

Finally, IVIVC and the relevance of T cell assays were reported by Hamze et al. (21). The authors identified CD4+ T cell epitopes in rituximab and infliximab using T cell and MAPPs assays and healthy donor cells and observed that two thirds of these epitopes were found to activate T cells in the peripheral blood of treated patient who had developed ADA. To our knowledge, the use of CD4+ T cell assay results as predictive markers of ADA development during clinical trials has not been reported.

Different assay formats have been used to assess the immunogenic potential of biologics. The basic formats are whole PBMC assays, with or without CD8+ depletion, and DC:T cell assays (**Table 1**). In principle, all assays rely on a source of professional APCs that are cocultured with T cells. Whole PBMCs assays rely on various APCs to process the biologic and present biologic-derived epitopes to CD4+ T cells. Conversely, DC:T cell assays require the isolation and differentiation of monocytes into immature monocyte-derived DCs (moDCs) as the sole source of APCs, which are incubated with the test article and matured with activation cocktails. Subsequently, mature loaded moDCs are washed and cocultured with autologous CD4+ T cells. MoDCs are highly efficient at processing antigens and presenting potential epitopes to CD4+ T cells. On the other hand, whole PBMC assays are easier to set up, more time- and cost-efficient and represent a more complete set of immune cells than DC:T assays. Critically, these cannot be used for biologics with immunomodulatory properties as this could mask the inherent sequence-derived immunogenicity potential of a test article. Next to testing the full protein, a whole PBMC assay, with or without CD8+ depletion, is also used to screen biologic-derived peptides, selected by previous *in silico* or MHC-associated peptide proteomics (MAPPs) analyses (22), or peptide libraries overlapping the whole protein sequence. These assays add granularity to the assessment, allowing the identification of high risk CD4+ T cell epitopes within the protein sequence, opening the possibility of a deimmunization strategy.

Various readouts can be used to assess test article-induced CD4+ T cells responses in any assay format (whole PBMCs, CD8+-depleted PBMCs or DC:T assays). Proliferation analysis has been widely applied, originally based on ^3^H-thymidine incorporation (23), although newer flow cytometry-based methods (e.g. CFSE (24, 25), EdU (24), Ki-67 staining (26) have since been developed that allow the specific detection of proliferation within the CD4+ T cell population without the need of radioactive reagents or specialist lab infrastructure. Other readouts include the detection of IL-2 (23) or IFN-γ secretion via ELISpot or FluoroSpot (24, 27–30), the assessment of activation induced markers (AIM) by flow cytometry (26) or the detection of IFN-γ, TNF-α and IL-2 by flow cytometry intracellular cytokine staining (31). Note that this list is not exhaustive and does not represent all the assay formats and readouts used within the community of immunogenicity experts.

## Section 3 - Terms and definitions

A comprehensive list of terms and definitions related to ADA, including distinctions such as incidence versus prevalence and persistent versus transient ADA, has been developed by industry leaders (32, 33) for a consistent description and interpretation of ADA responses across clinical studies. Similarly, there is a need to align on terms and definitions for immunogenicity-related activities that take place prior to clinical development. **Table 2** comprises the terms and definitions established by the EIP NCIRA group. We clarified the phrase “non-clinical immunogenicity risk assessment” as the assessment of risks related to immunogenicity with non-clinical experiments and existing data. This includes *in silico* tools, *in vitro* assays and preclinical animal studies. The non-clinical work supports risk assessment during preclinical and clinical development, especially related to product-related risk factors. Animal studies can inform the potential consequences of ADA on drug pharmacokinetics and safety, which might guide the design of the clinical monitoring and mitigation plan especially when there is high conservation of an endogenous protein candidate with high sequence homology. The NCIRA informs the risk of developing ADA with more limited capability to inform the potential consequences of ADA, which depends on many factors. Clarity on terminology is fundamental to successful application of best practices in non-clinical immunogenicity risk assessment, as these serve as a guiding framework to drive the development of robust assays and facilitate accurate data interpretation. In the case of *in vitro* T cell assays, inclusion of several controls which interrogate critical assay variables such as cell viability, responsiveness, and non-specific activation is paramount to ensure correct data interpretation. The specific role of each control in the assay, together with some examples, are detailed in section 5. Additionally, a comprehensive characterization of the assay would be conducted to determine the degree of suitability of the assay for its different use cases (e.g., decision-making processes for lead candidate selection, *post-hoc* analyses, submission to regulatory agencies). Contrary to ADA assays, the validation process of non-clinical immunogenicity risk assessment assays is not delineated in immunogenicity guidelines. To avoid any confusion, the term “fit-for-purpose qualification” is recommended regarding characterization of those assays. This encompasses, but is not limited to the interrogation of critically relevant parameters such as accuracy, precision, detection limit, linearity, sensitivity and specificity (17, 34).

**Table 2:** Terms and definitions.

Terms and definitions	
Term	Definition
Non-clinical immunogenicity risk assessment	Assessment of risks related to immunogenicity with non-clinical experiments and existing data. Includes *in silico* tools, *in vitro* assays and preclinical animal studies. Supports risk assessment during clinical product development, especially related to product-related risk factors. Informs the risk of developing ADA with limited capability to inform the potential consequences of ADA.
Baseline response control	Assess cell responses induced in absence of any non-specific stimulus or drug
Confounder control	Evaluate cell responses triggered by drug components that are not the focus but could interfere with the T cell response assessment
Cell functionality control	Assess cell functionality and responsiveness, i.e., ensure cells in the assays are capable to develop a response to an appropriate strong stimulus
Sensitivity control	Assess the ability of the assay to detect responses to test articles with a range of magnitude.
Assay qualification	The qualification might include assessment of accuracy, precision, detection limit, linearity, robustness, sensitivity and specificity

## Section 4 - Donor selection and cell quality

### Section 4.1 - Donors

The selection of donors for a T cell assay depends on several factors including the donor set size, the desired human leukocyte antigen (HLA) coverage for the assay, and pre-defined inclusion and exclusion criteria. The number of donors directly influences the sensitivity to detect a minimal frequency of responses **Table 3**. The chance of detecting a sample showing no signal depends on the frequency of responses in the population and on the sample size. Therefore, the size of the donor set is ideally determined statistically based on the preliminary risk assessment of the product (35). Factors such as the degree of humanness, the number of mutations, molecular format, and linkers are considered. The lower the anticipated response frequency, the more donors are necessary to have confidence in flagging the risk of immunogenicity. In the authors’ experience, donor sets of 30–50 donors are usually implemented for T cell assays to assess immunogenicity, depending on the estimated risk level for the molecules to be tested.

**Table 3:** Donor selection and cell quality.

Donor selection and cell quality	
Category	Details
Donor selection	
Cohort size	The size of donor set is determined statistically, based on the anticipated immunogenicity potential of the product assessed as part of immunogenicity risk assessment (degree of humanness, number of mutations, molecular format, linkers) The lower the expected response, the more donors are necessary to have confidence in the frequency of responding donors
Exclusion criteria	Parameters used as exclusion criteria (for example age, disease, vaccination status, ongoing infections) should be specified
Inclusion criteria	Donors should exhibit diversity in age, gender, ethnicity and most importantly in HLA genetic background
HLA allele coverage	If the donors’ HLA haplotypes are not accessible, the donor set should be increased above the calculated minimum number of donors (usually 50 donors are used in such a case) If HLA haplotypes are available, the donor set should exhibit an HLA allele distribution representative of the population(s) of interest, e.g., intended clinical trial cohorts Allele distributions can also be matched for HLA II supertypes (closely related HLA alleles with conserved binding pockets despite genetic variability). The combination of supertypes to match should be chosen to ensure coverage of the population of interest Ideally, HLA-DP and HLA-DQ representativity should also be taken into consideration, but the feasibility of the approach will depend on access to a large pool of donors to select from
Cell quality	
Blood collection and PBMC isolation	Time from blood draw to PBMC isolation should ideally not exceed 6 hours Establish standard operating procedures for PBMC freezing and thawing
Viability	Assess cell viability via flow cytometry as soon as possible after blood withdrawal, or cell thawing if using cryopreserved samples Consider removal of dead cells using a commercial kit Reject samples if the viability of the population of interest (T cells or subsets of T cells, DCs) is less than 90% at the start of the experiment or the analyzed T cell population is below 80% at the end of the experiment
Cell content and phenotype	Assess cell composition and phenotypes by flow cytometry: • Ratio between B and T cells • Frequency of APCs • Content of naïve vs memory CD4+/CD8+ T cells (CD45RA, CD45RO) • Content of regulatory T cell (Tregs) (CD25, CD127, or intracellular staining for FoxP3) • Activation state: • Expression levels of CD69, CD40L on T cells • Expression levels of CD83, CD86, CD40, HLA-DR on APCs
Cell functionality	Functionality/responsiveness of cells should be tested using a cell functionality control (see **Table 4**) Samples that fail to develop a response to the functionality control should be excluded from the final analysis, unless the functionality control used is KLH: some protocols do not exclude KLH non-responders

Regarding the inclusion of the appropriate donors, ideally the donor set exhibits diversity in age, gender, ethnicity, and genetic background mostly based on HLA haplotype, to ensure that different populations are represented in the assay. If the donors’ HLA haplotypes are not accessible (e.g., regulation, cost), the number of donors should exceed the minimum number calculated based on the assay qualification.

As HLA molecules exhibit different peptide binding motifs, different sets of therapeutic-derived potential T cell epitopes might be presented across donors. Albeit not the only factor determining the development of a T cell response, allele coverage is an essential consideration for extrapolating T cell assay results to a population of interest. If HLA haplotypes are available, the donor set should ideally reflect the HLA allele distribution of the target population. Frequencies of the populations of interest can be calculated using the allele frequency net database (36). Algorithms to match HLA types of donors to a preferred patient population have been developed such as the one reported by McGill et al. (37). Alternatively, donor set selection can be based on the coverage of HLA-DR HLA II supertypes. Each HLA supertype includes closely related HLA alleles with conserved binding pockets, despite genetic variability. Examples of combinations of supertypes used to represent different target populations have been described (38–41).

Most commonly, the donors’ haplotypes are matched to those of the target population for HLA-DRB alleles, mainly DRB1, with emerging importance of DRB3, DRB4 and DRB5 (42). However, it is advisable to consider HLA-DP and HLA-DQ representation when feasible. This may depend on the availability of a large pool of donors from which to select. Exceptionally, a T cell assay can be run with a set of specifically identified donors if the expectation is that the factors in those donors are suitable for the question to be answered. For example, if a product becomes available to a new population enriched for a specific HLA type, then a skewing of the assay donors would be appropriate to assess the T cell immunogenicity risk.

Regarding the exclusion of inappropriate donors, these criteria should be clearly defined and may include factors such as age, disease, vaccination status, and ongoing infections. These parameters ensure that donors who might skew the results are excluded from the set.

### Section 4.2 - Cells

Controlling the quality of the cells used for T cell assays is critical to obtain reliable data. The key parameters to be controlled are cell viability, cell functionality, cell subsets (nature and number), and phenotype. The time from blood draw to PBMC isolation has a significant impact on viability and functionality of T cells. Based on the group’s experience, the time should ideally not exceed 6 hours to maximize the quality of the PBMCs. When using cryopreserved PBMCs, it is recommended to establish a standard operating procedure for freezing (covering parameters such as freezing medium, number of cells per vial, the rate of cooling, short-term and long-term storage) and thawing (for instance thawing rate, medium, washes, recovery). Cell viability should be assessed as soon as possible after blood withdrawal or thawing of frozen PBMC samples. Dead cells may be removed using commercially available kits.

Samples should be excluded if the viability of the target populations (e.g., T cells, subsets of T cells, dendritic cells (DCs)) is less than 90% at the start of the experiment or the analyzed T cell population upon assay completion is below 80%. The functionality and responsiveness of cells should be tested using a cell functionality control (see **Table 4** for details). Samples failing this quality control will be excluded from the final analysis. The natural biological variation in immune cell composition and activation phenotypes can be large and must be carefully assessed using flow cytometry. Once an assay has been established and qualified, in the author’s experience, tracking the following parameters can be helpful to identify anomalous, particularly false negative, results: CD4+/CD8+ T cell ratio, B and T cells ratio, APC nature and frequency, naïve versus memory CD4+/CD8+ T cells (CD45RA, CD45RO) content, regulatory T cells content (CD25, CD127, or FoxP3), activation state (CD69 and CD40L on T cells; CD83, CD86, CD40, and HLA-DR on APCs). For flow cytometry endpoints, it is recommended to collect at least 5,000 events. Higher numbers of events could increase assay sensitivity, with this parameter being investigated during qualification.

**Table 4:** Reagents and material quality.

Reagents and material quality		
Category	Details	Examples
Lot testing and bridging experiments	Should be performed on critical reagents and material used as functionality and sensitivity controls	Critical reagents Medium; medium supplements such as fetal bovine serum (FBS); antibodies; cytokines Functionality and sensitivity controls (see examples in **Table 5**)
Impurities	Endotoxin level should be below 0.1-0.5 EU/mg or up to 1 EU/mg for material in early development stages Host cell proteins (HCPs)^1^: specific HCPs may modulate DC maturation and confound T cell assay results. As testing all drug candidates for individual HCPs by default is impractical, unexpectedly strong signals in the T cell assay could trigger HCP identification and further investigation	HCPs with reported adjuvant activity include for example Flagellin, RPS3, HMGB1, PRDX1, S100A4, PLBL2, HSP70 (43)
Generic peptides	Definition applied by FDA: max. 40 amino acids. In T cell assays, the generic peptide as such is tested and compared to the different impurities the drug product contains	For the 5 molecules mentioned in the guideline glucagon, liraglutide, nesiritide, teriparatide, and teduglutide: clear guidelines from FDA For the others: product-specific guidances (PSGs) -> Not all peptides need (the same) immunogenicity potential assessment
Short synthetic peptides (derived from longer peptides or proteins sequences)	Justify the choice of length and overlap when scanning a whole protein sequence. For instance, longer peptides might better represent flanking residues but do not allow for exact binding core determination Peptide purity should be ≥95% where feasible. If not, the nature of contaminants (length or sequence variants) should be established Peptide solubility should be assessed in final resolubilized individual or pooled peptide material via mass spectrometry to avoid insoluble peptides giving false results in the T cell assay.	Commonly used lengths: 15-21-mers Purity ≥95%

^1^Health authorities have not defined acceptable thresholds of HCPs but generally recommend minimizing them as much as possible. Apart from determination of overall HCP levels, individual HCPs in drug substance and drug product are typically not identified during drug development by default but only in case of elevated levels in late stages of a program.

The robustness of these selection and quality control criteria ensures high confidence in the outcomes of experiments and ensures the reproducibility and accuracy of the research findings.

## Section 5 - Reagent and material quality

The quality control of materials is essential for the establishment of a reliable assay. Critical reagents of the assay and test materials should meet suitable acceptable pre-defined criteria to ensure a valid outcome. Reagent lot testing and bridging experiments, together with thorough qualitative analysis of test material are recommended and presented in **Table 4**.

For the test materials, impurities should be understood and controlled as far as practical. The purity of the material during drug design might not be as high as later in development. For example, endotoxin is known to interfere with cell proliferation while host cell proteins can stimulate T cells raised against them. It is therefore important to measure them as far as practicable and to limit their amount. The consensus for endotoxin levels is to not exceed 0.5 EU/mg (survey conducted with NCIRA members), although up to 1 EU/mg for materials in early development stages is acceptable if associated with data scrutiny to identify any consequent interference in the assay. Host cell proteins (HCPs) can modulate DC maturation, i.e. suppress DC maturation or act as adjuvants, and can prime HCP-specific T cells, leading to an incorrect estimation of the T cell response to the drug. While it is impractical to test all drug candidates for individual HCPs by default, overall HCP levels should be as low as possible, and in case of unexpectedly strong signals in the T cell assay, HCP identification and further investigation may be warranted. Examples of HCPs with reported adjuvant activity have been summarized previously (43). Health authorities have not defined acceptable thresholds of HCPs but generally recommend minimizing them as much as possible and in general recommend a risk-based evaluation of potential immune responses to impurities, with a testing program designed accordingly (44, 45). Apart from the determination of overall HCP levels, individual HCPs in drug substances and drug products are typically not identified during drug development by default but only in the case of elevated overall HCP levels in late stages of a program. Where data is available, we recommend reporting them as a proportion of test article (e.g. in parts per million). It is worth noting that individual HCPs have not been characterized in T cell assays, despite predicted and observed risks for PLBL2 (46). In one study, mAbs tested with approximately 4000 ppm and up to 134 different HCP species did not add significantly to T cell responses (47).

Short peptides designed for *in vitro* testing from longer peptides or protein sequences should be carefully selected. Selection can be done based on *in silico* analyses and/or *in vitro* MHC associated peptide presentation analyses. If the election is based on overlapping peptides spanning the whole sequence of the protein, positive T cell responses should be interpreted with caution as without additional analyses it remains unclear whether this peptide can be presented naturally. The length and overlap of peptides for identification of T cell epitopes should be justified when designing peptide libraries. While longer peptides might better represent flanking residues, they do not allow for exact binding core determination. Common lengths used for short peptides are 15-21-mers. Synthetic peptide purity should be ≥95% where feasible. If purity is lower, it is recommended to determine whether the contaminants are length variants or sequence variants. In the case of length variants, the concentration in the assay should be adjusted to ensure that the correct quantity of the test peptide is added, i.e., based on the percentage of contaminants. If considerable amounts of sequence variants are present in the peptide preparation, assay results may not be interpretable. Most commonly however, the nature of the contaminants is not known and peptides with purity <95% should be re-synthesized, re-purified, or analyzed considering the potential for a spurious response. A lower purity of peptide may be acceptable in a screening assay, accepting the risk of false positive or false negative results. Peptide solubility should be assessed in final resolubilized individual or pooled peptide material via mass spectrometry to avoid insoluble peptides giving false results in the T cell assay.

This detailed quality control process ensures that reagents and materials used in experiments will provide reliable results, contributing to the accuracy and reproducibility of assay data and correct interpretation.

## Section 6 - Assay controls

T cell assays are complex biological assays and therefore need additional controls beyond those expected for biochemical or biophysical assays which are used to measure other aspects of product quality or developability during non-clinical development of biotherapeutics. Essential controls for T cell assays interrogate cell functionality, specificity of the response, and assay sensitivity and are presented in **Table 5**.

**Table 5:** Assay controls.

Assay Controls		
Category	Details	Examples
Cell functionality control	Assess cell functionality and responsiveness, i.e., ensure T cells in the assay are capable to develop a response to an appropriate strong specific or non-specific stimulus	Strong antigen-specific naïve responses: Keyhole limpet hemocyanin (KLH) Non-specific responses: anti-CD3 and anti-CD28 antibodies; Phytohemagglutinin (PHA) Of note, staphylococcal enterotoxin B (SEB) should be used with caution as aerosols could contaminate neighboring wells
Baseline response control	Assess cell responses induced in absence of stimulus	Reconstitution medium, e.g. DMSO for synthetic peptides Relevant buffer/formulation Medium alone
Confounder control	Concern complex modalities Evaluate cell responses triggered by drug components that are not the focus of but could interfere with the T cell response assessment	Cytokines for cytokine fusion proteins Vector alone, cargo alone for products comprising a vector and a cargo (e.g., mRNA-LNPs)
Sensitivity controls	Assess the ability of the assay to detect responses to test articles with a range of donor response frequencies Biologically relevant sensitivity controls should: • Where possible, be of the closest format and length as test articles i.e., protein, short peptides, longer peptides • For short peptides, individual peptides are preferred as peptide pools tend to give larger responses. Pairs of control peptides can be used to cover required HLA breadth • Induce a naïve or recall response based on the type of donor material used: healthy donors (naïve) or treated-patient samples (recall) • Exhibit the anticipated donor response frequencies or represent a range between lower and higher responses be commonly used, chosen based on own experience or the scientific literature	Short synthetic peptides: Single peptide with promiscuous HLA binding *Naïve responses:* therapeutic-derived peptides validated *ex vivo* (CD4+ T cell epitopes from e.g., rituximab, infliximab and natalizumab); PADRE (48) in assays with rounds of restimulation *Recall responses:* Peptides from viruses most donors will have been exposed to (infection or vaccination, e.g., HLA II-restricted CEFT, i.e. peptides derived from human Cytomegalovirus (CMV), Epstein-Barr Virus (and Influenza Virus (Flu) and Tetanus Toxoid (TT); HLA I-restricted CEFT peptides) Whole proteins: *Naïve responses:* Therapeutics commonly used in *in vitro* assays such as ATR-107 (high T cell response frequencies), bococizumab (high T cell response frequencies), bevacizumab (low T cell response frequencies) and natalizumab (intermediate T cell response frequencies) *Recall responses:* same as for peptide controls but whole protein format e.g., tuberculin purified protein derivative (PPD), tetanus toxoid (TT), Influenza virus hemagglutinin (Flu HA) Generic peptides: reference product, lixisenatide (high T cell response frequencies), salmon calcitonin (high T cell response frequencies)

^1^KLH is highly immunogenic but its size makes it irrelevant as a sensitivity control.

### Section 6.1 - Cell functionality control

The cell functionality control assesses the ability of the cells to act in response to non-specific or strong naïve antigen addition. There are two main types of functionality controls. The first category comprises compounds that assess T cell functionality only. These are non-specific T cell activators, which directly stimulate T cell activation/proliferation. These include phytohemagglutinin (PHA), a plant lectin that binds to surface glycoproteins including T cell receptors, agonist antibodies such as anti-CD3 and anti-CD28 used separately or in combination, and Staphylococcal Enterotoxin B (SEB), a superantigen which binds both the TCR and the costimulatory receptor CD28 on T cells as well as MHC class II and CD80, CD86 molecules on APCs, triggering polyclonal T cell activation and cytokine release (49–51). However, SEB should be used with precautionary measures to limit aerosols, which may contaminate neighbouring wells in standard assay formats. The lack of response to T cell activators indicates that T cells are not functional and therefore should lead to the exclusion of the donor from the analysis. The magnitude of response to the functionality control indicates the maximum response the T cells are capable of and can be used to understand differences in donor responses to the tested drugs for a particular assay. Maximum responses can be obtained using the combination of antibodies in a captured format, e.g. immobilized on beads, to establish a signalling complex mimicking the immunological synapse. The second cell functionality control category comprises compounds that assess the functionality of both APC and T cells, i.e., the steps leading to and including T cell activation. These are highly immunogenic proteins capable of inducing a naïve T cell response, such as Keyhole Limpet hemocyanin (KLH) used in several published T cell immunogenicity assays (17, 52–54). The response to KLH may vary depending on the assay format. In a DC:T assay, unlike non-specific T cell activators, KLH will not induce a response in 100% of the donors. Hence, two scenarios with different donor exclusion outcomes might be considered: 1) If a donor does not respond to KLH nor any test articles, it is impossible to tell whether the response to the test articles represent true negative responses (KLH non-responsive donor) or if it is due to cells that are not functional. In this case, exclusion of the donor is recommended; 2) Conversely, if a donor does not respond to KLH but a response to the sensitivity control is observed, the donor can be retained as the cells have proven functional. A more conservative approach has been described for some assays, where no response to KLH suffices to exclude a donor (52). Building a cell bank of KLH-responding donors could be considered to reduce donor attrition in such studies.

### Section 6.2 - Baseline response control

The baseline response control assesses the cell response induced in the absence of a stimulus, which commonly arises from donor-to-donor variability in immune system activation status, cell handling procedures prior to and within the assay, and through the necessary assay components distinct from the drug. Medium, buffer, or drug formulation of the drug are suitable baseline response control. In the case of synthetic peptides designed from longer peptides (40 amino acids and above) or protein sequences for *in vitro* testing, reconstitution components such as dimethyl sulfoxide (DMSO), at the equivalent concentration as found in the test sample, would be suitable. Of note, based on the group’s experience, DMSO will induce toxicity if such concentrations were to exceed 0.5% and therefore should be avoided. An internal body of data for each assay should be generated to determine the assay-specific threshold above which a donor will be excluded from the final analysis.

### Section 6.3 - Confounder controls

A confounder control evaluates T cell responses triggered by a drug component that may not contribute sequences with potential to be T cell epitopes, but interferes with the assay, usually through enhancing or directly stimulating T cell activation and/or proliferation. This concerns components of complex modalities such as the cytokine payload for antibody-cytokine fusion proteins or the vector and the cargo alone for products comprising a vector and a cargo, e.g. mRNA-LNP particles (55, 56).

### Section 6.4 - Sensitivity controls

The sensitivity controls assess the ability of the assay to detect responses to test articles over the dynamic range of the assay. These controls are biologically relevant molecules with known assay response profiles, meaning that sufficient data exists to define an expected range of response, either for the proportion of donors responding or the magnitude of the response. Ideally, the response profiles of these sensitivity controls have been established in the specific assay in which they are used. They may be whole proteins or peptides and could be therapeutic agents or known immunostimulators. T cell assays are increasingly being used with samples from treated patients, therefore sensitivity controls for both naïve and recall responses should be considered. Sensitivity controls are particularly critical to demonstrate the ability of the assay to detect naïve T cell responses.

Based on published literature and our own experience of running and applying assays to make decisions in early drug development, we recommend that suitable sensitivity controls have the closest format and length to the test articles, i.e. proteins consistent with therapeutic scaffold where possible (for instance such controls might not yet be available for recent novel modalities), and peptides of short and longer lengths. Furthermore, we recommend inclusion of at least two sensitivity controls that exhibit the expected donor response frequencies and represent the lower and higher end of the responses. Comparison of test articles to benchmarks at the same concentrations is desirable. For monoclonal antibodies and other biologics with higher molecular weight, optimal assay results are typically observed within a concentration range of 0.3 to 4 µM. This range is based on the collective experience of members of the EIP-NCIRA working group. In contrast, peptides are commonly tested at higher molarities, up to 10 µM. These benchmarks help with the interpretation of results if they are from a set of commonly used molecules or defined standards.

In the case of assessing short peptides, we recommend including individual control peptides rather than peptide pools, which have the potential to exhibit higher responses than single peptides through a larger coverage of HLA alleles and/or wider TCR repertoire recruitment. Suitable high T cell response controls for short peptides should be single peptides with a broad spectrum of binding to different HLA types (promiscuous binders) to obtain responses from a maximum number of donors based on the broad range of HLA types included in the experimental set. If a single peptide does not achieve this goal, two peptides can be used instead but tested separately.

For naïve responses, peptides derived from therapeutic peptides that have been shown to elicit T cell responses in healthy donors or patients can be used. These include CD4+ T cell epitopes from e.g., rituximab, infliximab, natalizumab and adalimumab as well as the HLA II promiscuous binder PADRE for assays with several rounds of restimulation (26, 30, 57, 58). For recall responses, peptide pools from pathogens most donors have been exposed to such as cytomegalovirus (CMV), Epstein-Barr virus (EBV), Influenza (Flu) and tetanus toxoid (TT), have been developed both for HLA class I and class II responses and are often used (59–63). Using HLA II-restricted peptides from these pools separately is recommended when determining individual test article peptides, although sensitivity control peptide pools are acceptable as comparators if test article peptides are also pooled.

Care should be taken in assessing the T cell responses to whole proteins due to a possible impact of the mechanism of action (MoA) of the protein on the assay readout (see section 7.3). Combined APC-T cell assays may overcome some of these limitations as the excess protein that has not been taken up by APCs is washed away before coculture with T cells. Several potential sensitivity controls for assessing whole protein naïve responses are available based on reports of T cell assay responses in conjunction with experience of ADA incidence and impact. The most used therapeutics include the anti-IL21 receptor ATR-107 and the anti-PCSK9 bococizumab as high T cell response sensitivity controls (34), and the anti-VEGF bevacizumab as low T cell response sensitivity control (15). The anti-a4 integrin natalizumab, which shows responses in fewer donors than the high sensitivity controls might be included as additional benchmark (21). For recall responses to whole proteins, pathogen-derived proteins are favored, including tuberculin purified protein derivative (PPD), TT, and Flu hemagglutinin (Flu HA) (64–66). When comparing biosimilars or generic peptides, inclusion of the reference product is recommended. For peptide products longer than 40 amino acids, lixisenatide and salmon calcitonin are possible high sensitivity controls (23, 67).

## Section 7 - Data analysis

There is a considerable amount of data generated with each T cell assay, depending on the variety and number of endpoints measured and at what frequency. Here we aim to provide guidance on the analysis of key assay results to maximize the confidence in decision making processes. These key assay outcomes are the response of individual donors to each test article, the magnitude of this response, and, for each test article, the total number of donors that exhibit a positive response, i.e., the donor response frequency **Table 6**.

**Table 6:** Data analysis.

Data analysis	
Parameter	Details
Donor positive response to a test article	The signal-to-noise threshold for a response to be positive should be determined statistically The degree of confidence associated with the cut-off should be specified
Donor response frequency to a test article	Can be used to rank-order molecules and estimate an immunogenicity potential relative to the sensitivity controls Donor response frequency differences between compounds should be interrogated statistically and the degree of confidence should be specified
Donor response magnitude to a test article	Can indicate donor-specific factors that influence the response and might not be detected with the baseline control. Statistical analysis should include relative responsiveness of the donor across all tests to identify hyper- or hypo-responsiveness.

Determining whether a donor’s response to a test article is positive requires careful statistical analysis. A crucial component of this analysis is establishing a signal-to-noise threshold. This threshold serves as a cut-off point, distinguishing genuine immune responses from background noise, which is inherent to biological assays. The process involves statistical methods that set this cut-off, ensuring that only responses above the noise threshold are considered positive. Furthermore, specifying the degree of confidence associated with this cut-off is essential. By defining this confidence level, researchers can ensure clarity and reproducibility in their results, thereby enhancing the reliability of their conclusions.

The frequency with which donors respond to a test article provides valuable information for ranking molecules and estimating their relative immunogenicity potential in comparison to sensitivity controls. This measure assesses how frequently a response can be detected in the donor set when exposed to the test article compared with a control substance. Statistical interrogation of response frequency differences between various compounds is necessary to draw meaningful comparisons. By quantifying these differences, researchers can gain a robust understanding of the comparative immunogenicity of the test compounds. This analysis helps in identifying which molecules elicit broader immune responses and may thus bear a higher immunogenic potential. Of note, some statistical tests are not adequate to determine if molecules have significant differences in donor response frequencies. For instance, approaches to set thresholds or determine positivity that assume normal distribution of data (e.g. mean + k*SD or t-test) are not correct, as T cell proliferation data is not normally distributed. These approaches can be used after appropriate transformation of the data. Using an empirical threshold from literature, which is not based on performance of the assay, should also be avoided.

Analyzing the magnitude of donor responses to a test article can reveal additional levels of immune stimulation beyond just response positivity. A higher magnitude response may suggest the presence of immune interactions specific to the donor such as a higher number of T cell precursors in the assay, or an increased overall activation in the presence of test articles that could not be detected with the baseline control. To interpret these findings accurately, statistical analysis should encompass an evaluation of each donor’s responsiveness across all test articles. This comprehensive analysis helps identify donors who are hyper-responsive or hypo-responsive, thereby ensuring that variations in response are attributed to the test articles rather than individual outliers in the donor set.

Incorporating rigorous statistical methods and defining degrees of confidence during data analysis are crucial steps in accurately interpreting T cell assay response data. These practices enable the effective ranking of test articles, identification of significant immunological activities, and distinction between true signals and background noise.

In the near future, analysis of T cell assay data is expected to be improved with technological advances to enable generation of larger data sets and their interpretation. Machine learning models could assist with interpreting T cell assay responses in relation to genetic data, including HLA analysis and other contributing factors. A learning cycle, incorporating systems immunology tools such as TCPro (68) would contribute to improvements in assays and the overall contributions of these assays to the immunogenicity risk assessment.

## Section 8 - Additional considerations

The complexity of T cell assays often translates into numerous protocol iterations to develop an assay that exhibits the desired characteristics. Once established, assay execution for a given project requires careful attention to detail as described in the previous sections. To further increase confidence in the data, the group suggests to also take into consideration the following points:

### Section 8.1 - Qualification

Conducting qualification of the assay is recommended to ascertain its accuracy, precision, detection limit, linearity, robustness, sensitivity and specificity. Such fit-for-purpose qualification have been reported and could be used to design one’s own (17, 34). Establishing positivity and difference thresholds is critical for interpreting results accurately and consistently. Considering all these factors can enhance the robustness of experimental findings, facilitate data interpretation, and increase the impact of the assay on candidate selection and/or overall program immunogenicity risk assessment (IRA), and in return, the development of effective therapies.

### Section 8.2 - Cell survival

Ensuring the survival of a sufficient number of cells of interest until the end of the assay is critical for accurate and reliable results. This can be particularly challenging in longer assays, where cell viability may decline over time. It may be beneficial to add cytokines such as IL-2 and IL-7 to the culture medium if these cytokines are not measured as part of the readout. Cytokines can support cell survival and proliferation, thereby maintaining the quality of the assay. If cell viability falls below 80% at the end of a prolonged assay using supplemented serum-free medium, consider whether the addition of FBS or AB serum to the culture could enhance cell survival. Addition of cytokines and serum components should be carefully evaluated to ensure they do not alter the baseline response levels, which could confound the experimental results per condition, and are adequate to detect the desired responses.

### Section 8.3 - Mechanism of action

In protein-based T cell assays, the drug’s MoA must be considered, as it could lead to misinterpretation of the data. MoAs that modulate T cell responses directly (e.g. T cell engaging bispecific antibodies blinatumomab, mosunetuzumab, tarlatamab, epcoritamab, teclistamab, and immune checkpoint inhibitors pembrolizumab, nivolumab and ipilimumab) or indirectly; expression of target on APCs (69), and/or interference with pathways involved in cell maturation, activation, or drug endocytosis. The need to isolate the impact of MoA and the intrinsic immunogenicity of the molecule might depend on the context in which the assay is being used. If the assay is conducted to compare candidates and select the lead with the most favourable potential T cell epitope risk profile, the T cell response should only reflect the T cell risk associated with it. Moreover, if a biotherapeutic is expected to reduce T cell responses, assay signals may be low, making candidate selection challenging. In the case of MoAs that act on T cells, a DC:T assay format where the drug does not get in contact with T cells would be chosen. If the MoA impacts the DCs, one could consider that this impact will be present in all conditions and therefore the assay would still test the potential T cell epitope risk.

If the protein-based T cell assay is used to assess the risk of the final candidate, one could consider including the MoAs to access the overall T cell risk that result from the sequence in presence of the MoA. In the case of immunosuppressant MoA, the low or no T cell response in the assay could be considered representative of the *in vivo* situation (drug sequence, T cells, APCs, MoA at play), that is, that a low T cell response means a lower risk of ADA development even in presence of CD4+ T cell epitopes. However, a translational understanding of the extent that the MoA would dampen the T cell responses and its consequences on ADA development might not be known. In the case of immunostimulators, it is recommended to assess the potential T cell epitope risk and mitigate it by design, as non-specific amplification of the T cell response might result in activation of T cells specific for lower immunogenicity risk peptides that would not otherwise induce a detectable response.

If a whole PBMC assay format is chosen, one should ensure that the T cell responses remain within the assay’s dynamic range. For this, the drug could be spiked in the sensitivity controls to evaluate synergistic or antagonistic effects.

Lastly, the potential for some therapeutics to deplete specific cell populations should be considered; for instance, B cell depletion in whole PBMC assays can reduce the number of APCs present in the assay.

### Section 8.4 - Therapeutic modality

The T cell assays reviewed here can be applied to assess risks related to specific sequences in isolation or in the context of the whole molecule. In addition to the mechanism of action considerations above, protein therapeutics may interact differently in T cell assays due to interactions with immune receptors. For antibody-based therapeutics, consideration should be given to potential interactions with Fc receptors. For proteins or peptides with endogenous counterparts an assessment of interaction with immune cells helps to determine how the therapeutic might perform in the different assay formats, either in the case of a receptor on an immune cell specific for the protein or interaction through a binding partner. Additional consideration should be given to structural variants, e.g. different glycosylation patterns, that could contribute to immune cell uptake or activation.

### Section 8.5 - Response kinetics

Assays may feature various readouts, such as activation marker expression, cytokine secretion, and cell proliferation. For each individual activation marker, it is essential to determine the appropriate measurement time-point based on the kinetics of the response in the assay. Properly timed measurements can ensure that data collected reflects the true nature of the immune response, especially when negative responses are observed.

### Section 8.6 - Short peptide design

The number of CD4+ T cell epitopes predicted by *in silico* algorithms solely based on binding affinity might be high and their testing costly and labour intensive. An approach to reduce the number of short peptides (15 mers) to test in a PBMC assay for instance, is to establish *in vitro* which of these peptides are presented by APCs (31, 70–73). The most appropriate *in vitro* assay for this is the MAPPs assay, that identifies the fragments of the molecule presented at the surface of APCs after internalization and antigen processing (74). Using accurate and relevant data sources to guide peptide design can enhance the assay’s ability to identify significant epitopes, as there may be limitations to the number of available cells and conditions that can be tested.

### Section 8.7 - Regulatory landscape

The therapeutic area where these assays are recommended by the US Food and Drug Administration (FDA) is the generic peptide field, for which specific Abbreviated New Drug Application (75) and Product-Specific Guidance for Generic Drug Development (76) exist. For these submissions *in vitro* assays are frequently used to demonstrate equivalence between a generic peptide and its respective reference-listed drug. A rigorous qualification report for the chosen assay should be included in the study results, demonstrating that the assay is fit-for-purpose and meets all quality criteria, including the responses to the various controls outlined in this paper. In contrast to date, inclusion of *in vitro* immunogenicity data for therapeutic proteins, gene and cell therapy products, as well as oligonucleotide-based biologics data in Marketing Authorization Application (MAA, European Union) and Biologic License Applications (BLA, United States) dossiers is not a regulatory requirement. Nevertheless, it has become more frequent for sponsors to incorporate candidate drug sequence-related risk assessment in IND dossiers, which in majority consist of *in silico* CD4+ T cell epitope prediction only or accompanied by one whole protein T cell proliferation *in vitro* assay. In the biosimilar space, during an FDA Interim Public Meeting of the Biosimilar User Fee Act (BsUFA) III Regulatory Science Program, J. Resiliac and K. E. Howard presented a poster titled “*In Vitro* Immunogenicity Assay Submissions in Biosimilar Drug Applications” (77). This data mining study revealed that sponsors frequently included *in vitro* immunogenicity assays to support clinical trial data in biosimilar applications (351(k)), although not a regulatory requirement. The assays reported included cytokine release, mixed lymphocyte reaction (MLR), and T cell proliferation (DC:T). However, the study also highlighted substantial variability in assay types, protocols, and cell sources, which complicate data interpretation. These findings, which apply to therapeutic proteins and other non-generic peptide biologics underscore the need for harmonized best practices to improve the interpretability and regulatory utility of such data in immunogenicity risk assessment.

## Section 9 - Discussion

Immune responses to biological therapies continue to confound development of new medicines. Applying an understanding of immune response mechanisms and known risk factors for biologics – from peptides, through proteins to gene therapies enables the assessment and mitigation of risk during the design phase. Although no single assay enables the prediction of immunogenicity risk for biologics (2), several assays have emerged to enable differential categorization of risk for lead molecules, or for the assessment of product-related risk for inclusion in the overall immunogenicity risk assessment (78) Several variations of T cell assays have been developed to identify lower immunogenicity risk molecules by assessing whether peptides from new molecules can be both presented to and recognized by T cells likely to help B cells develop clinically relevant anti-drug antibody responses (79).

Application of the T cell assays is complicated by the multiple formats of assays that have been designed for specific contexts. The complexity of these assays makes standardization challenging, and it is unlikely that all laboratories in industry and academia use identical protocols. It is more realistic to encourage harmonization of assays across the industry, which is needed to improve interpretation of data across the expanding range of biologics in development and will help identify when specific differences in assay format are justified for specifics contexts of use. The European Immunogenicity Platform recommends best practices for experimental design, assay conditions, and data analysis to reduce analytical variability, facilitate data interpretation, and provide confidence in the data obtained from T cell assays.

T cell assays utilize fresh or frozen PBMCs most commonly from healthy donors. Donor to donor variability has been reported as the most significant source of assay variability (17). Under certain circumstances, PBMCs from patients with a relevant disease could be assessed, although there are no published reports of this in practice. The limited supply of PBMCs from an individual donor at each donation complicates the establishment of biobanks of reproducible donor sets. Recalling local donors or sourcing large quantities of PBMCs from Leukopaks aims to mitigate these issues. Furthermore, the current recommendation is to include a donor set that covers diverse HLA types that may represent either a global population, or matches the ethnicity of the anticipated phase 1 population. Dependent on the program, inclusion of HLA-types representative of different potential patient populations should be considered. Of potential significance for population diversity, a focus on canonical HLA types might be insufficient for identifying the genetic risk for immunogenicity, with non-canonical HLA SNPs associated with CD4+ binding regions (80) and cytokine SNPs (81, 82) reported as contributing to immunogenicity outcomes. There are no reports of approaches to include genetic diversity beyond HLA type into these T cell assays.

A concern with the current application of these T cell assays is the presumed insufficient number of T cells, and therefore proportion of the TCR repertoire, surveyed from each donor. Published assays survey from 1 x 10^5^–5 x 10^6^ T cells per donor (**Table 1**). However, analysis of the frequency of T cells capable of recognizing sequences from biologics suggests that the frequency of specific T cells range between 0.15 and 1 cells per million, often with undetectable levels in the majority of donors with assays that have a lower limit of quantification of 0.15 cells per million (15, 83). This example would suggest that a high number of T cells per donor might be needed to truly assess whether that donor can generate a T cell response. Therefore, this working group recommends 2 million T cells per sample as a reasonable compromise between sensitivity and practicality. The limited availability of specific T cells is likely a contributing factor as to why immunogenicity scientists report the apparent lack of association between specific HLA type and response in T cell assays. This phenomenon would also explain the donor response frequency threshold of 10% reported to correlate with ADA incidences greater than 20% and the rarity of high frequency responses for more immunogenic proteins (17). The authors commonly observe donor response frequencies of 20–30% for the more immunogenic proteins in the DC:T assay format, rising to 40% and above in a PBMC assay format (23). These factors contribute to the need for a higher number of donors required to identify differences between molecules, especially during candidate selection where the leads might show variation in single amino acids. Future research could help identify the optimal cell numbers for different applications, for example early screening of leads would likely need higher throughput, which might be easier with more donors rather than more cells, whereas an assay to assess the risk for a chosen candidate may benefit from surveying higher cell numbers to give more confidence of a translatable result.

Correlating T cell assay results with clinical ADA incidence values for specified sensitivity controls is often done to demonstrate the value the assay adds to the overall assessment. However, T cell responses are one of many contributing factors to the induction of ADA and might not necessarily correlate directly with clinical ADA incidence (2). Correlations between assay outcome and the ADA incidence of sensitivity controls indicate the relative level of T cell activation only. It is not realistic to expect that T cell assays alone can predict clinical immunogenicity due to the complexity of the immune response and the limited variables that can be tested *in vitro*.

Assay controls are essential for establishing confidence in an assay during development and to ensure that each run delivers believable results. EIP uses the term ‘control’ to denote molecules that help understand assay performance for each run. This is preferred to the term ‘standard’, which implies a consistent response outcome that is not possible with the donor-to-donor variability that occurs in these T cell assays. One effort that is currently ongoing to address the question of controls to enable harmonization of T cell assay result interpretation between different laboratories, is an initiative funded by the Health and Environmental Sciences Institute Immuno-Safety Technical Committee in collaboration with the National Institute for Biological Standards and Control (NIBSC) to generate a reference panel of biotherapeutics (with known low, mid or high immunogenicity incidence in the clinic) manufactured in a standard and qualitative manner and that can be used as sensitivity controls across laboratories. Similarly, FDA is leading an effort to build a reference panel for assays that assess innate immune response modulating impurities (IIRMIs) immunogenic risk, in collaboration with the National Institute of Standards and Technology (NIST, US Department of Commerce) and several companies testing the candidate controls. These common sources and the use of reference material would address a key component of assay harmonization.

Application of the T cell assays to product development depends on both the confidence in the chosen assay being able to answer the questions being asked of it, and the time taken to generate data from the assay. As discussed, context of use is important in establishing whether T cell assays are used to address screening questions, are focused on ranking and/or selection of leads, are contributing to the overall immunogenicity risk assessment prior to clinical trials, are being applied to process changes, or are surveying clinical trial subjects. This context of use will help identify the practical use of complementary assays and their relative timing. For example, if a T cell assay were to be run in very early project stages to identify peptides with immunogenic potential, then *in silico* HLA binding prediction tools would be used to focus on the peptides most likely to produce a response, whereas the MAPPs assay to show presented peptides might not be possible due to time or material constraints. Other risk factors are included in the overall risk assessment, so conditions that contribute to different potential immunogenicity outcomes, e.g. MOA, target on APCs, restimulation, etc., should be considered in the setup of the T cell assays.

The accuracy of *in silico* tools used for the prediction of HLA-peptide binding, i.e. potential T cell epitopes, has improved dramatically since 2019, by integrating large-scale MAPPs assay (elution) datasets and refining machine learning algorithms (70, 71). However, the state of the art *in silico* models still face challenges, e.g. in form of limited training data for HLA-DP and HLA-DQ peptide binding and the fact that they are only predicting peptide binding according to the amino acid sequence of the biotherapeutic and are not accurately considering biotherapeutic uptake and processing. Thus, they are currently best positioned to be used in early development phases, where not enough material for *in vitro* approaches is available and to inform the design of confirmatory *in vitro* experiments but cannot yet fully replace them. *In silico* prediction of specific T cell responses, i.e. TCR recognition, is even harder than prediction of HLA-peptide binding. AI/ML approaches predicting TCR recognition show relatively high prediction accuracy on test datasets containing training data, but struggle with new, unseen test sets (84). To enhance the prediction accuracy of these tools, integrating advanced structural predictions of the TCR: HLA-peptide complex and the significant expansion of the training dataset will be necessary.

A future goal of immunogenicity research is to establish translatable models of the complete immune response, expanding the DC:T type assays to include activation and subsequent maturation of B cells to produce anti-drug antibodies representative of those occurring in clinical trial subjects and patients. Currently, most B cell assays for assessing immunogenicity are run independently of the T cell assays and are used to assess the presence of biotherapeutic-specific memory B cells responses in treatment naïve subjects. B cells can be differentiated into antibody secreting cells (ASCs) followed by an evaluation for biotherapeutic-specific ASCs (29, 85). Complex culture systems have been developed to model the interaction between T and B cells that take place within the germinal centre. Various 2D and 3D *in vitro* and *ex vivo* models are available, including organoids (86). Human tonsillar organoids have been shown to exhibit antibody class switching, affinity maturation, and antigen-specific somatic hypermutation of human B lymphocytes against pathogen and vaccine antigens. These *in vitro* models could therefore provide relevant information on the risk/potential for biologic-specific ADA generation. However, further research is required to improve these models and their translational value in predicting immunogenicity risk/potential of biologics. At present, B cell responses in the context of immunogenicity risk assessment are not commonly assessed and not standardized.

EIP recommends the publication of assay details to improve comparison of current assays and improve the harmonization of assays for specific contexts of use. Incremental improvements in assays could overcome challenges in translatability of the T cell assays if the context of use is understood. An example of this is the need for assessing chronic exposure to help predict the outcomes of longer-term trials or real-world application of therapies as the full extent of immunogenicity is often not seen during the limited exposure in clinical trials. With biologics generally being relatively weak antigens, these assays may not be sufficiently sensitive to detect responses after the initial stimulation or a single restimulation of the biologic. However, maintaining suitable culture conditions for additional restimulations is currently not possible. Furthermore, publication of additional data relating to product quality attributes will be useful in determining the value of currently available assays to support process developments (47, 87–89).

In conclusion, the current range of assays and application of those assays makes comparison across published literature difficult. The relatively small data sets published for each assay tend to show some correlation with clinical ADA incidences, which may not accurately reflect the ability of these assays to predict clinical ADA for new molecules. Confidence in the T cell assays for decision making during drug design and development together with regulatory acceptance of the assays will be improved with harmonized, controlled assays. A better understanding of the impact of assay variables will assist harmonization of data sets for automated analyses through application of AI. For now, the T cell assays should be used with careful thought about the context of use and how the outcomes can be used for decision making at each stage of development.
